# Robust Decentralized Nonlinear Control for a Twin Rotor MIMO System

**DOI:** 10.3390/s16081160

**Published:** 2016-07-27

**Authors:** Lidia María Belmonte, Rafael Morales, Antonio Fernández-Caballero, José Andrés Somolinos

**Affiliations:** 1 Escuela de Ingenieros Industriales de Albacete, Universidad de Castilla-La Mancha, 02071 Albacete, Spain; LidiaMaria.Belmonte@uclm.es (L.M.B.); Antonio.Fdez@uclm.es (A.F.-C.); 2 Escuela Técnica Superior de Ingenieros Navales, Universidad Politécnica de Madrid, 28040 Madrid, Spain; joseandres.somolinos@upm.es

**Keywords:** decentralized control, nonlinear control, time-scale model, Euler–Lagrange model, TRMS

## Abstract

This article presents the design of a novel decentralized nonlinear multivariate control scheme for an underactuated, nonlinear and multivariate laboratory helicopter denominated the twin rotor MIMO system (TRMS). The TRMS is characterized by a coupling effect between rotor dynamics and the body of the model, which is due to the action-reaction principle originated in the acceleration and deceleration of the motor-propeller groups. The proposed controller is composed of two nested loops that are utilized to achieve stabilization and precise trajectory tracking tasks for the controlled position of the generalized coordinates of the TRMS. The nonlinear internal loop is used to control the electrical dynamics of the platform, and the nonlinear external loop allows the platform to be perfectly stabilized and positioned in space. Finally, we illustrate the theoretical control developments with a set of experiments in order to verify the effectiveness of the proposed nonlinear decentralized feedback controller, in which a comparative study with other controllers is performed, illustrating the excellent performance of the proposed robust decentralized control scheme in both stabilization and trajectory tracking tasks.

## 1. Introduction

In the last few years, there has been an increased interest from researchers in developing control algorithms for unmanned aerial vehicles (UAVs) [1,2,3,4,5,6], due to the multiple applications and uses of this type of vehicle. This has motivated the use of new laboratory platforms capable of simulating the operation of the UAVs. This way, it is possible to perform experimental tests for evaluating the different designs developed. We can highlight the three-DOF hover system [7], the three-DOF helicopter system [8] and the twin rotor MIMO system (TRMS) [9], which is the platform used in this research.

**Figure 1:**
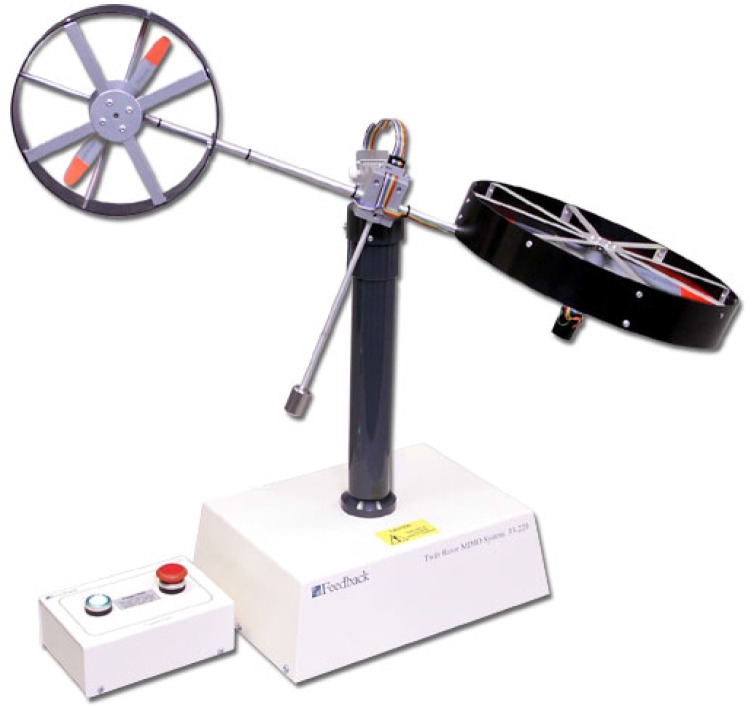
Twin rotor MIMO system (TRMS).

The TRMS is a nonlinear and multivariate laboratory helicopter specifically designed to test and evaluate control algorithms by means of the MATLAB/Simulink^®^ software environment. The dynamic behavior of the system is similar to a real helicopter, but with some differences due to the construction of the model that greatly hinder the modeling and design of control algorithms for this platform. As can be seen in Figure 1, the TRMS is formed by a base attached to a tower, at which end is a two-dimensional pivot that allows the mobile structure to rotate freely. The mobile part is composed of two metal beams: the horizontal beam in which ends the main and tail rotors with the corresponding propellers are positioned in perpendicular planes and the counterbalance beam affixed to the horizontal beam at the pivot to move the equilibrium point of the system.

The electrical part of the TRMS is mainly composed of two DC motors that drive the propellers of both rotors and the interface circuit, an internal electrical circuit that adapts the input control voltages, applied in MATLAB/Simulink^®^, to the actual voltage value applied to each DC motor. Thus, a change in the control voltages produces a variation in the supply voltages of the motors, which results in a variation of the rotational speed of each propeller, measured by a tachometer. This way, a change in propulsive forces finally results in the movement of the platform. The movement, in the vertical and horizontal planes, is measured by two encoders that determine the pitch and yaw angles, respectively.

The movement of the TRMS presents not only a high cross-coupling between the two rotors as in a real helicopter, but also a coupling effect between rotor dynamics and the body of the model. This is due to the action-reaction principle originated in the acceleration and deceleration of the motor-propeller groups. Therefore, the control system of the TRMS generates a significant difference with regard to a real helicopter by varying the voltages applied to the rotors, which greatly complicates the system dynamics. On the other hand, the TRMS is also an underactuated system as a result of fewer control actions, which are the voltages applied to the respective rotors, compared to the four degrees of freedom of the system, which are: the pitch and yaw angles and the angular velocities of the propellers. Moreover, there are many physical parameters that cannot be measured exactly, and some of the parameters supplied by the manufacturer are changed by time, such as the friction coefficients. All of this makes the modeling and control of the system a difficult task to achieve.

There are many research works that have addressed this challenging experimental platform. In fact, the dynamic modeling of the TRMS has been studied from different approaches. Rahideh et al. define the dynamic model of the TRMS using Newtonian and Lagrangian methods and also by means of two models based on neuronal networks using Levenberg–Marquardt (LM) and gradient descent (GD) algorithms [10]. Toha et al. develop a parametric model for the TRMS based on dynamic spread factor particle swarm optimization [11]. A linear parameter varying (LPV) method of identification, by taking a local approach, is considered in order to derive an LPV model for TRMS by means of interpolation and approximation in the work of Tanaka et al. [12]. The Euler–Lagrange method is employed in the research of Tastermirov [13] to obtain a complete dynamic model of the TRMS, which is tuned and validated experimentally. More recently, a model based on first-principle modeling and later improved by gray box modeling, has been presented in [14]. The design of control algorithms for the TRMS platform has been also investigated via several approaches and control methods. Among the different contributions in this area, we can cite the following works. Juang and colleagues present a comparative study [15], by means of numerical simulations, between classical control schemes, based on the Ziegler–Nichols proportional-integral-derivative (PID) rule, the gain margin and phase margin rule, the pole placement method and novel controllers based on fuzzy logic and genetic algorithms. In the research of Wen et al. [16], the dynamic model for the TMRS is decoupled into two single input single output (SISO) systems in order to apply a PID-based robust deadbeat control scheme for each of them, thus achieving the control of the platform. The design and experimental validation of a multi-step Newton-type model predictive control (MPC) to control the TRMS is presented in [17] where a nonlinear dynamic model of the platform is also developed. An adaptive fuzzy controller to stabilize the TRMS in a desired position or to track a specified trajectory is discussed in [18]. The work of Pandey et al. [19] in which two conventional PID controllers, improved by the use of derivative filter coefficients, are employed to the control of the pitch and yaw angles, the work of Belmonte et al. based on active disturbance rejection control (ADRC) [20] and the research of Alagoz et al. [21] about a reference model-based optimization approach for the online auto-tuning of PIDs using the stochastic multi-parameters divergence optimization (SMDO) method are other interesting investigations that are focused in the design of control schemes for the TRMS.

In the particular case of this research, we present the design of a novel decentralized nonlinear controller for the TRMS, composed of two control loops in a cascade scheme. The development of the proposed control scheme has been separated into two independent stages: the design of the inner loop or electrical controller, which is used to control the angular velocity of each propeller, and the design of the outer loop or mechanical controller, which is employed to determine the necessary velocities to control the space position of the TRMS. The effectiveness of the proposed scheme is validated by means of the experiments performed in the laboratory platform in which the proposed nonlinear controller shows an excellent performance for both stabilization and tracking tasks.

The rest of the article is organized as follows: Section 2 introduces the dynamic model of the TRMS, showing the modeling of the electrical part formed by the interface circuit and the DC motors, and the modeling of the mechanical part composed by the equations of motion of the system. Next, the design of the proposed decentralized control scheme is detailed in Section 3. The experiments carried out in order to verify the efficiency of the proposed control algorithm are presented in Section 4, where we detail the experimental setup and the obtained results, which include a comparative study with other classical controllers. Finally, some conclusions are provided in Section 5.

## 2. Dynamic Model

This section describes the dynamic modeling of the TRMS, and according to [10], it has been divided into the following two stages. In the first place, the electrical part of the platform is modeled, including the interface circuit, the DC motors and the propulsive forces produced by these motors. Then, a Lagrangian-based model is employed for the remaining mechanical structure. Next, each part of the dynamic model is dealt with in the next subsections.

### 2.1. Dynamics of the Electrical Part

The main and tail rotors (denominated as *m* and *t*, respectively) are assumed to be identical with different mechanical loads. The mathematical expressions governing the main and tail rotors are the following:

- Main rotor:
  (1) $$\begin{array}{ccc} L_{m}\frac{di_{m}}{dt} & = & v_{m}-k_{v_{m}}\omega_{m}-R_{m}i_{m} \end{array}$$
  (2) $$\begin{array}{ccc} I_{m_{1}}{\dot{\omega }}_{m} & = & k_{t_{m}}i_{m}-f_{v_{m}}\omega_{m}-C_{Q_{m}}\omega_{m}\left|\omega_{m}\right| \end{array}$$
- Tail rotor:
  (3) $$\begin{array}{ccc} L_{t}\frac{di_{t}}{dt} & = & v_{t}-k_{v_{t}}\omega_{t}-R_{t}i_{t} \end{array}$$
  (4) $$\begin{array}{ccc} I_{t_{1}}{\dot{\omega }}_{t} & = & k_{t_{t}}i_{t}-f_{v_{t}}\omega_{t}-C_{Q_{t}}\omega_{t}\left|\omega_{t}\right| \end{array}$$

where $i_{m}$ and $i_{t}$ are the main and tail motor currents, respectively, $L_{m}$ and $L_{t}$ represent the motor inductances, $R_{m}$ and $R_{t}$ denote the motor resistances, $k_{v_{m}}$ and $k_{v_{t}}$ express the motor back electromotive force (EMF) constants, $\omega_{m}$ and $\omega_{t}$ are the angular velocities of the propellers and $v_{m}$ and $v_{t}$ represent the input voltage of the DC motors. $I_{m_{1}}$ and $I_{t_{1}}$ define the moment of inertia of the rotors; the terms $k_{t_{m}}i_{m}$ and $k_{t_{t}}i_{t}$ express the main and tail electromechanical torques generated by the DC motors; $C_{Q_{m}}\omega_{m}\left|\omega_{m}\right|$ and $C_{Q_{t}}\omega_{t}\left|\omega_{t}\right|$ illustrate the aerodynamic torques; and $f_{v_{m}}\omega_{m}$ and $f_{v_{t}}\omega_{t}$ denote the friction torques. Following a similar argument as [13], the dynamics of the current of the motors defined in Expressions (1) and (3) is ignored due to the higher value of the DC motor mechanical time constants against the electrical ones. In fact, the DC motor mechanical constants ($c_{m_{m}}$ and $c_{m_{t}}$) are in the order of $10^{3}$-times higher than the DC motor electrical constants ($c_{e_{m}}$ and $c_{e_{t}}$), as you may observe in Table 1, which shows the parameters of both rotors. Thereby, for the DC motor circuits, the following algebraic equations are obtained:

(5) $$v_{m}-k_{v_{m}}\omega_{m}-R_{m}i_{m}=0$$

(6) $$v_{t}-k_{v_{t}}\omega_{t}-R_{t}i_{t}=0$$

It should be noted that the magnitude input voltages of the main and tail rotors in the MATLAB/Simulink^®^ environment, defined as $u_{m}$ and $u_{t}$, respectively, and the motor terminal voltages, defined as $v_{m}$ and $v_{t}$, respectively, are nonlinear (the signals pass through a circuit interface), as was demonstrated in [10]. In our developments, it is assumed that the relationship between the control signals and the motor voltages is linear and that the differences will be canceled at the controller stage. Therefore, the relationships between the control signals and the MATLAB/Simulink^®^ environment are the following:

(7) $$\begin{array}{ccc} v_{m} & = & k_{u_{m}}u_{m} \end{array}$$

(8) $$\begin{array}{ccc} v_{t} & = & k_{u_{t}}u_{t} \end{array}$$

in which $k_{u_{m}}$ and $k_{u_{t}}$ are defined as constant gains. Upon operating with Equations (1)–(8) and rearranging terms, the following two equations are yielded for the main and rail rotors of the TRMS:

(9) $$\begin{array}{ccc} {\dot{\omega }}_{m} & = & \frac{k_{t_{m}}k_{u_{m}}}{I_{m_{1}}R_{m}}u_{m}-\left(\frac{k_{t_{m}}k_{v_{m}}}{R_{m}}+f_{v_{m}}\right)\frac{\omega_{m}}{I_{m_{1}}}-\frac{C_{Q_{m}}}{I_{m_{1}}}\omega_{m}\left|\omega_{m}\right| \end{array}$$

(10) $$\begin{array}{ccc} {\dot{\omega }}_{t} & = & \frac{k_{t_{t}}k_{u_{t}}}{I_{t_{1}}R_{t}}u_{t}-\left(\frac{k_{t_{t}}k_{v_{t}}}{R_{t}}+f_{v_{t}}\right)\frac{\omega_{t}}{I_{t_{1}}}-\frac{C_{Q_{t}}}{I_{t_{1}}}\omega_{t}\left|\omega_{t}\right| \end{array}$$

in which the value and units of each parameter of the main and tail rotors are detailed in Table 1. Finally, if we use matrix notation, the dynamic model of the electrical part of the TRMS can be expressed by means of the following expression:

(11) $$\dot{\omega }\left(t\right)=Nu\left(t\right)+\Gamma \left(\omega \left(t\right)\right)$$

where $\omega \left(t\right)={\left[\omega_{m}\left(t\right),\omega_{t}\left(t\right)\right]}^{T}$ is the angular velocity vector, $u\left(t\right)={\left[u_{m}\left(t\right),u_{t}\left(t\right)\right]}^{T}$ is the input control voltage vector and, finally, the diagonal positive matrix $N=diag(n_{m},n_{t})$ and the vector $\Gamma \left(\omega \left(t\right)\right)={\left[\Gamma_{m}\left(t\right),\Gamma_{t}\left(t\right)\right]}^{T}$ are given by:

(12) $$N=\left[\begin{array}{cc} n_{m} & 0\\ 0 & n_{t} \end{array}\right]=\left[\begin{array}{cc} \frac{k_{t_{m}}k_{u_{m}}}{I_{m_{1}}R_{m}} & 0\\ 0 & \frac{k_{t_{t}}k_{u_{t}}}{I_{t_{1}}R_{t}} \end{array}\right]$$

(13) $$\Gamma \left(\omega \left(t\right)\right)=\left[\begin{array}{c} \Gamma_{m}\left(t\right)\\ \Gamma_{t}\left(t\right) \end{array}\right]=\left[\begin{array}{c} -\left(\frac{k_{t_{m}}k_{v_{m}}}{R_{m}}+f_{v_{m}}\right)\frac{\omega_{m}}{I_{m_{1}}}-\frac{C_{Q_{m}}}{I_{m_{1}}}\omega_{m}\left|\omega_{m}\right|\\ -\left(\frac{k_{t_{t}}k_{v_{t}}}{R_{t}}+f_{v_{t}}\right)\frac{\omega_{t}}{I_{t_{1}}}-\frac{C_{Q_{t}}}{I_{t_{1}}}\omega_{t}\left|\omega_{t}\right| \end{array}\right]$$

### 2.2. Dynamics of the Mechanical Part

If the developments reported in [10] are used as a basis, the dynamics of the TRMS can be derived using Lagrange’s formulation:

(14) $$\frac{d}{dt}\left(\frac{\partial L}{\partial \dot{q}}\right)-\frac{\partial L}{\partial q}=Q$$

where L=K−V is the Lagrangian function, *K* and *V* are the kinetic and potential energies of the TRMS, $q\left(t\right)={\left[\psi \left(t\right),\phi \left(t\right)\right]}^{T}$ is a vector of generalized coordinates and $Q\left(t\right)={\left[Q_{\psi }\left(t\right),Q_{\phi }\left(t\right)\right]}^{T}$ denotes the vector of generalized forces in the TRMS. All of the necessary terms of (14) are obtained in the following subsections.

#### 2.2.1. Evaluation of the Kinetic Energy

**Figure 2:**
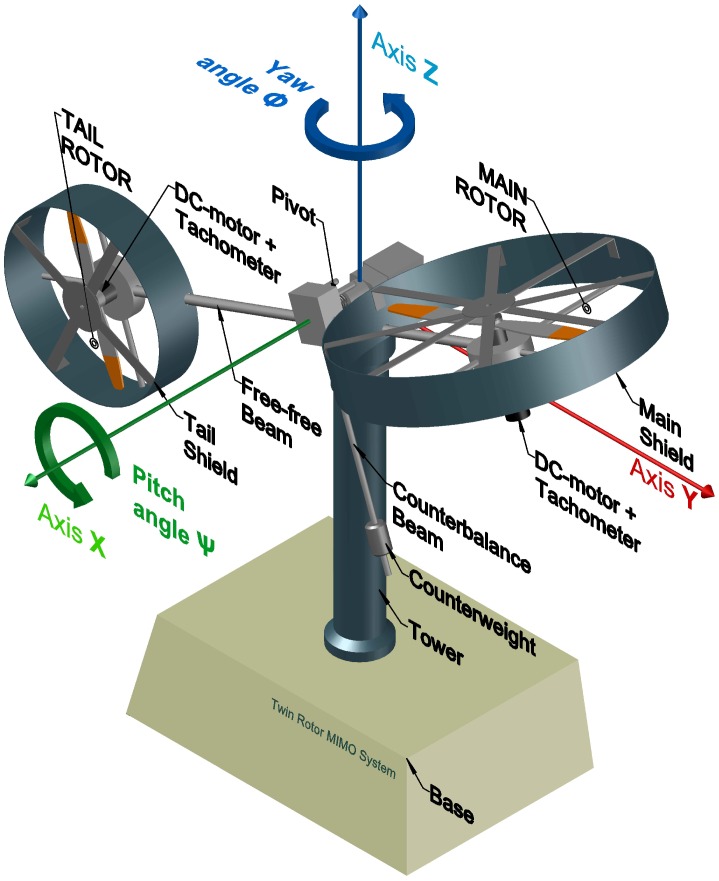
Twin rotor MIMO system (TRMS) prototype platform.

**Figure 3:**
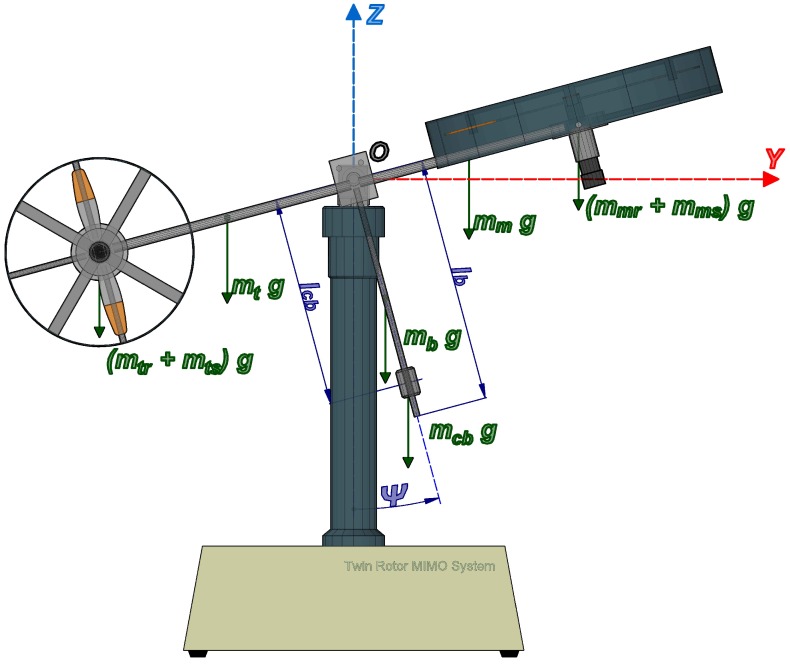
View of the TRMS on the vertical plane.

**Figure 4:**
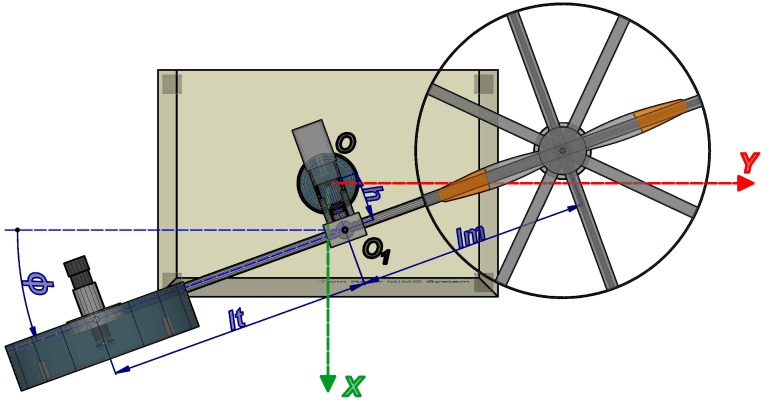
View of the TRMS on the horizontal plane.

In order to calculate the energy of the TRMS, we consider the platform as divided into the following three subsystems: (1) the subsystem composed of the free-free beam (tail and main beam), tail rotor, main rotor, tail shield and main shield; (2) the counterbalance beam with the counterweight; and (3) the pivoted beam (see Figure 2, Figure 3 and Figure 4). The positions of the subsystems can be expressed as the position of a point for each one, $P_{1}$, $P_{2}$, $P_{3}$, parametrized by the distance between it and the point where the subsystem can rotate, as can be observed in the following expressions (where $S_{\psi }\equiv \sin\psi$, $C_{\psi }\equiv \cos\psi$, $S_{\phi }\equiv \sin\phi$ and $C_{\phi }\equiv \cos\phi$):

(15) $$\begin{array}{ccc} P_{1}\left(R_{1}\right) & = & {\left[\begin{array}{ccc} P_{1_{x}} & P_{1_{y}} & P_{1_{z}} \end{array}\right]}^{T}={\left[\begin{array}{ccc} -R_{1}S_{\phi }C_{\psi }+hC_{\phi } & R_{1}C_{\phi }C_{\psi }+hS_{\phi } & R_{1}S_{\psi } \end{array}\right]}^{T} \end{array}$$

(16) $$\begin{array}{ccc} P_{2}\left(R_{2}\right) & = & {\left[\begin{array}{ccc} P_{2_{x}} & P_{2_{y}} & P_{2_{z}} \end{array}\right]}^{T}={\left[\begin{array}{ccc} -R_{2}S_{\phi }S_{\psi }+hC_{\phi } & R_{2}C_{\phi }S_{\psi }+hS_{\phi } & -R_{2}C_{\psi } \end{array}\right]}^{T} \end{array}$$

(17) $$\begin{array}{ccc} P_{3}\left(R_{3}\right) & = & {\left[\begin{array}{ccc} P_{3_{x}} & P_{3_{y}} & P_{3_{z}} \end{array}\right]}^{T}={\left[\begin{array}{ccc} R_{3}C_{\phi } & R_{3}S_{\phi } & 0 \end{array}\right]}^{T} \end{array}$$

where $R_{1}$ and $R_{2}$ are the distances from point $O_{1}$ to $P_{1}$ and $P_{2}$, respectively, and $R_{3}$ is the distance from $P_{3}$ to the center of the reference system, that is the point *O*.

In this way, the total amount of kinetic energy consists of the sum of the following three terms:

(18) $$K=\sum_{i=1}^{3}K_{i}=\frac{1}{2}\sum_{i=1}^{3}\int v_{i}^{2}\left(R_{i}\right)dm\left(R_{i}\right)$$

where $K_{i}$ denotes the kinetic energy of each subsystem and $v_{i}\left(R_{i}\right)$ is the velocity of each subsystem parameterized by $R_{i}$, which represents the distances $R_{1}$, $R_{2}$ and $R_{3}$ that have been defined above. The calculations of these energies are the following:

(19) $$\begin{array}{ccc} K_{1} & = & \frac{1}{2}J_{1}\left(C_{\psi }^{2}\dot{\phi^{2}}+\dot{\psi^{2}}\right)+\frac{1}{2}h^{2}m_{T_{1}}\dot{\phi^{2}}-hS_{\psi }l_{T_{1}}m_{T_{1}}\dot{\phi }\dot{\psi } \end{array}$$

(20) $$\begin{array}{ccc} K_{2} & = & \frac{1}{2}J_{2}\left(S_{\psi }^{2}\dot{\phi^{2}}+\dot{\psi^{2}}\right)+\frac{1}{2}h^{2}m_{T_{2}}\dot{\phi^{2}}+hC_{\psi }l_{T_{2}}m_{T_{2}}\dot{\phi }\dot{\psi } \end{array}$$

(21) $$\begin{array}{ccc} K_{3} & = & \frac{1}{2}J_{3}\dot{\phi^{2}} \end{array}$$

where:

$$\begin{array}{ccc} J_{1} & = & m_{ts}r_{ts}^{2}+\frac{1}{2}m_{ms}r_{ms}^{2}+\left(\frac{1}{3}m_{t}+m_{tr}+m_{ts}\right)l_{t}^{2}+\left(\frac{1}{3}m_{m}+m_{mr}+m_{ms}\right)l_{m}^{2}\\ m_{T_{1}} & = & m_{m}+m_{mr}+m_{ms}+m_{t}+m_{tr}+m_{ts}\\ l_{T_{1}} & = & \frac{\left(\frac{m_{t}}{2}+m_{tr}+m_{ts}\right)l_{t}-\left(\frac{m_{m}}{2}+m_{mr}+m_{ms}\right)l_{m}}{m_{T_{1}}}\\ J_{2} & = & \frac{1}{3}m_{b}l_{b}^{2}+m_{cb}l_{cb}^{2}\\ m_{T_{2}} & = & m_{b}+m_{cb}\\ l_{T_{2}} & = & \frac{m_{b}\frac{l_{b}}{2}+m_{cb}l_{cb}}{m_{T_{2}}}\\ J_{3} & = & \frac{1}{3}m_{h}l_{h}^{2} \end{array}$$

#### 2.2.2. Evaluation of the Potential Energy

The total potential energy consists of the sum of the following three terms:

(22) $$V=\sum_{i=1}^{3}V_{i}=g\sum_{i=1}^{3}\int r_{zi}\left(R_{i}\right)dm\left(R_{i}\right)$$

where *g* denotes the gravity constant, $V_{i}$ represents the potential energy of each one of the three subsystems in which we have divided the platform and $r_{zi}\left(R_{i}\right)$ is the coordinate on the z-axis of the position of each subsystem ($P_{i_{z}}$). The calculation of these energies is as follows:

(23) $$\begin{array}{ccc} V_{1} & = & gS_{\psi }l_{T_{1}}m_{T_{1}} \end{array}$$

(24) $$\begin{array}{ccc} V_{2} & = & -gC_{\psi }l_{T_{2}}m_{T_{2}} \end{array}$$

(25) $$\begin{array}{ccc} V_{3} & = & 0 \end{array}$$

#### 2.2.3. Lagrangian

After substituting Expressions (18)–(25) in the Lagrangian expression, we obtain:

(26) $$\begin{array}{ccc} L=K-V & = & \frac{1}{2}\left(J_{1}C_{\psi }^{2}+J_{2}S_{\psi }^{2}+J_{3}+h^{2}\left(m_{T_{1}}+m_{T_{2}}\right)\right)\dot{\phi^{2}}+\frac{1}{2}\left(J_{1}+J_{2}\right)\dot{\psi^{2}}\\ & & +h\left(l_{T_{2}}m_{T_{2}}C_{\psi }-l_{T_{1}}m_{T_{1}}S_{\psi }\right)\dot{\phi }\dot{\psi }-g\left(l_{T_{1}}m_{T_{1}}S_{\psi }-l_{T_{2}}m_{T_{2}}C_{\psi }\right) \end{array}$$

#### 2.2.4. Generalized Forces

The external forces in the mechanical system are owing to the following four physical effects: (a) aerodynamic forces created by the propellers; (b) the electromechanical forces generated by the propellers; (c) the viscous forces that model the dissipative effects that are present in the system and; (d) the elastic force created by the cable. After grouping the effect of these forces for each generalized coordinate, the following result is achieved for $Q\left(t\right)={\left[Q_{\psi }\left(t\right),Q_{\phi }\left(t\right)\right]}^{T}$:

(27) $$\begin{array}{ccc} Q_{\psi }\left(t\right) & = & C_{T_{m}}\omega_{m}\left|\omega_{m}\right|l_{m}-C_{R_{t}}\omega_{t}\left|\omega_{t}\right|-\left(f_{v_{\psi }}\dot{\psi }+f_{c_{\psi }}sign\left(\dot{\psi }\right)\right)+k_{t}{\dot{\omega }}_{t} \end{array}$$

(28) $$\begin{array}{ccc} Q_{\phi }\left(t\right) & = & C_{T_{t}}\omega_{t}\left|\omega_{t}\right|l_{t}C_{\psi }-C_{R_{m}}\omega_{m}\left|\omega_{m}\right|C_{\psi }-\left(f_{v_{\phi }}\dot{\phi }+f_{c_{\phi }}sign\left(\dot{\phi }\right)\right)-C_{c}\left(\phi -\phi_{0}\right)\\ & + & k_{m}{\dot{\omega }}_{m}C_{\psi } \end{array}$$

where $C_{T_{m}}\omega_{m}\left|\omega_{m}\right|l_{m}$ and $C_{T_{t}}\omega_{t}\left|\omega_{t}\right|l_{t}C_{\psi }$ represent the aerodynamic thrust torques acting along the *ψ* and *ϕ* angles, respectively; $C_{R_{t}}\omega_{t}\left|\omega_{t}\right|$ and $C_{R_{m}}\omega_{m}\left|\omega_{m}\right|C_{\psi }$ denote the aerodynamic cross-couplings effects generated by the propeller; the terms $\left(f_{v_{\psi }}\dot{\psi }+f_{c_{\psi }}sign\left(\dot{\psi }\right)\right)$ and $\left(f_{v_{\phi }}\dot{\phi }+f_{c_{\phi }}sign\left(\dot{\phi }\right)\right)$ define the magnitudes of friction torques for each generalized coordinate; $k_{t}{\dot{\omega }}_{t}$ and $k_{m}{\dot{\omega }}_{m}C_{\psi }$ express the inertial counter torques that are owing to the reaction produced by a change in the rotational speed of the rotor propellers; and the term $C_{c}\left(\phi -\phi_{0}\right)$ is the magnitude of the torque exerted by the cable (it has a certain stiffness that allows us to model it as a spring) on the *ϕ* angle.

Finally, it should be noted that the works of Tastermirov et al. [13] and Mullhaupt et al. [22] provide more details about the external forces in the TRMS and other laboratory platforms with similar dynamics.

#### 2.2.5. Equations of Motion

Upon substituting Expressions (26)–(28) in Equation (14) and after some straightforward manipulations, we obtain the following equations of motion:

(29) $$\begin{array}{c} \left(J_{1}+J_{2}\right)\ddot{\psi }+h\left(l_{T_{2}}m_{T_{2}}C_{\psi }-l_{T_{1}}m_{T_{1}}S_{\psi }\right)\ddot{\phi }+\left(\frac{\left(J_{1}-J_{2}\right)}{2}S_{2\psi }\right){\dot{\phi }}^{2}+g\left(l_{T_{1}}m_{T_{1}}C_{\psi }+l_{T_{2}}m_{T_{2}}S_{\psi }\right)=\\ =C_{T_{m}}\omega_{m}\left|\omega_{m}\right|l_{m}-C_{R_{t}}\omega_{t}\left|\omega_{t}\right|-\left(f_{v_{\psi }}\dot{\psi }+f_{c_{\psi }}sign\left(\dot{\psi }\right)\right)+k_{t}{\dot{\omega }}_{t} \end{array}$$

(30) $$\begin{array}{c} h\left(l_{T_{2}}m_{T_{2}}C_{\psi }-l_{T_{1}}m_{T_{1}}S_{\psi }\right)\ddot{\psi }+\left(J_{1}C_{\psi }^{2}+J_{2}S_{\psi }^{2}+J_{3}+h^{2}\left(m_{T_{1}}+m_{T_{2}}\right)\right)\ddot{\phi }\\ -h\left(l_{T_{1}}m_{T_{1}}C_{\psi }+l_{T_{2}}m_{T_{2}}S_{\psi }\right){\dot{\psi }}^{2}+\left(\left(J_{2}-J_{1}\right)S_{2\psi }\right)\dot{\phi }\dot{\psi }=\\ =C_{T_{t}}\omega_{t}\left|\omega_{t}\right|l_{t}C_{\psi }-C_{R_{m}}\omega_{m}\left|\omega_{m}\right|C_{\psi }-\left(f_{v_{\phi }}\dot{\phi }+f_{c_{\phi }}sign\left(\dot{\phi }\right)\right)-C_{c}\left(\phi -\phi_{0}\right)+k_{m}{\dot{\omega }}_{m}C_{\psi } \end{array}$$

in which the value and units of all of the mechanical parameters are illustrated in Table 2 and Table 3, respectively. Finally, we can express the motion equations of the system in a compact form by means of matrix notation, thus obtaining the complete dynamic model of the mechanical part of the TRMS in the following expression:

(31) $$M\left(q\left(t\right)\right)\ddot{q}\left(t\right)+C\left(q\left(t\right),\dot{q}\left(t\right)\right)\dot{q}\left(t\right)+G\left(q\left(t\right)\right)+F\left(\dot{q}\left(t\right)\right)+T\left(q\left(t\right),\dot{\omega }\left(t\right)\right)=E\left(q\left(t\right)\right)\Omega \left(t\right)$$

where:

(32) $$M\left(q\left(t\right)\right)=\left[\begin{array}{cc} J_{1}+J_{2} & h\left(l_{T_{2}}m_{T_{2}}C_{\psi }-l_{T_{1}}m_{T_{1}}S_{\psi }\right)\\ h\left(l_{T_{2}}m_{T_{2}}C_{\psi }-l_{T_{1}}m_{T_{1}}S_{\psi }\right) & J_{1}C_{\psi }^{2}+J_{2}S_{\psi }^{2}+J_{3}+h^{2}\left(m_{T_{1}}+m_{T_{2}}\right) \end{array}\right]$$

(33) $$C\left(q\left(t\right),\dot{q}\left(t\right)\right)=\left[\begin{array}{cc} 0 & \frac{1}{2}\left(J_{1}-J_{2}\right)S_{2\psi }\dot{\phi }\\ -h\left(l_{T_{1}}m_{T_{1}}C_{\psi }+l_{T_{2}}m_{T_{2}}S_{\psi }\right)\dot{\psi } & \left(J_{2}-J_{1}\right)S_{2\psi }\dot{\psi } \end{array}\right]$$

(34) $$G\left(q\left(t\right)\right)=\left[\begin{array}{c} g\left(l_{T_{1}}m_{T_{1}}C_{\psi }+l_{T_{2}}m_{T_{2}}S_{\psi }\right)\\ 0 \end{array}\right]$$

(35) $$F\left(\dot{q}\left(t\right)\right)=\underset{F_{v}}{\underset{︸}{\left[\begin{array}{cc} f_{v_{\psi }} & 0\\ 0 & f_{v_{\phi }} \end{array}\right]}}\dot{q}\left(t\right)+\underset{F_{c}\left(\dot{q}\left(t\right)\right)}{\underset{︸}{\left[\begin{array}{c} f_{c_{\psi }}sgn\left(\dot{\psi }\right)\\ f_{c_{\phi }}sgn\left(\dot{\phi }\right) \end{array}\right]}}=\left[\begin{array}{c} f_{v_{\psi }}\dot{\psi }+f_{c_{\psi }}sgn\left(\dot{\psi }\right)\\ f_{v_{\phi }}\dot{\phi }+f_{c_{\phi }}sgn\left(\dot{\phi }\right) \end{array}\right]$$

(36) $$T\left(q\left(t\right),\dot{\omega }\left(t\right)\right)=\underset{M_{c}\left(q\left(t\right)\right)}{\underset{︸}{\left[\begin{array}{c} 0\\ C_{c}\left(\phi -\phi_{0}\right) \end{array}\right]}}-\underset{M_{i}\left(q\left(t\right)\right)}{\underset{︸}{\left[\begin{array}{cc} 0 & k_{t}\\ k_{m}C_{\psi } & 0 \end{array}\right]}}\dot{\omega }\left(t\right)=\left[\begin{array}{c} -k_{t}{\dot{\omega }}_{t}\\ C_{c}\left(\phi -\phi_{0}\right)-k_{m}{\dot{\omega }}_{m}C_{\psi } \end{array}\right]$$

(37) $$E\left(q\left(t\right)\right)=\left[\begin{array}{cc} C_{T_{m}}l_{m} & -C_{R_{t}}\\ -C_{R_{m}}C_{\psi } & C_{T_{t}}l_{t}C_{\psi } \end{array}\right]$$

(38) $$\Omega \left(t\right)=\left[\begin{array}{c} \omega_{m}\left|\omega_{m}\right|\\ \omega_{t}\left|\omega_{t}\right| \end{array}\right]$$

To conclude, the dynamic model of the mechanical part of the TRMS (31) can be summarized in a simplified form if we consider that the movement of the platform is sufficiently smooth. In this way, the terms of the inertial counter torques, $k_{t}{\dot{\omega }}_{t}$ and $k_{m}{\dot{\omega }}_{m}C_{\psi }$, can be considered negligible in comparison with the other terms. Thereby, the dynamic model of the TRMS can be rewritten as:

(39) $$M\left(q\left(t\right)\right)\ddot{q}\left(t\right)+D\left(q\left(t\right),\dot{q}\left(t\right)\right)=E\left(q\left(t\right)\right)\Omega \left(t\right)$$

where the matrices M(q(t)), E(q(t)) and Ω(t) have been defined in Equations (32), (37) and (38), respectively, and the new matrix $D\left(q\left(t\right),\dot{q}\left(t\right)\right)={\left[D_{\psi }\left(t\right),D_{\phi }\left(t\right)\right]}^{T}$ is given by:

(40) $$\begin{array}{ccc} D_{\psi }\left(t\right) & = & \frac{1}{2}\left(J_{1}-J_{2}\right)S_{2\psi }{\dot{\phi }}^{2}+g\left(l_{T_{1}}m_{T_{1}}C_{\psi }+l_{T_{2}}m_{T_{2}}S_{\psi }\right)+\left(f_{v_{\psi }}\dot{\psi }+f_{c_{\psi }}sgn\left(\dot{\psi }\right)\right) \end{array}$$

(41) $$\begin{array}{ccc} D_{\phi }\left(t\right) & = & -h\left(l_{T_{1}}m_{T_{1}}C_{\psi }+l_{T_{2}}m_{T_{2}}S_{\psi }\right){\dot{\psi }}^{2}+\left(\left(J_{2}-J_{1}\right)S_{2\psi }\right)\dot{\phi }\dot{\psi }+\left(f_{v_{\phi }}\dot{\phi }+f_{c_{\phi }}sgn\left(\dot{\phi }\right)\right)+C_{c}\left(\phi -\phi_{0}\right) \end{array}$$

## 3. Design of the Control System

The proposed decentralized nonlinear control scheme is based on decoupling the electrical dynamics from the mechanical dynamics. Once these dynamics have been decoupled, a nonlinear multivariate inner loop is closed in order to control the vector of the angular velocities of the propellers, $\omega \left(t\right)={\left[\omega_{m}\left(t\right),\omega_{t}\left(t\right)\right]}^{T}$, and then, a nonlinear multivariate outer loop is closed to control the vector of the generalized coordinates of the system, $q\left(t\right)={\left[\psi (t),\phi (t)\right]}^{T}$, in order to achieve stabilization and precise trajectory tracking tasks for the controlled position of the generalized coordinates of the TRMS. If we make the dynamics of the inner loop control much faster than the mechanical dynamics of the TRMS in Equation (39), the dynamics of the inner loop can be therefore made approximately equal to $I^{2\times 2}$, (i.e., $\omega^{*}\left(t\right)\approx \omega \left(t\right)$), and the outer loop can be designed independently [23].

**Figure 5:**
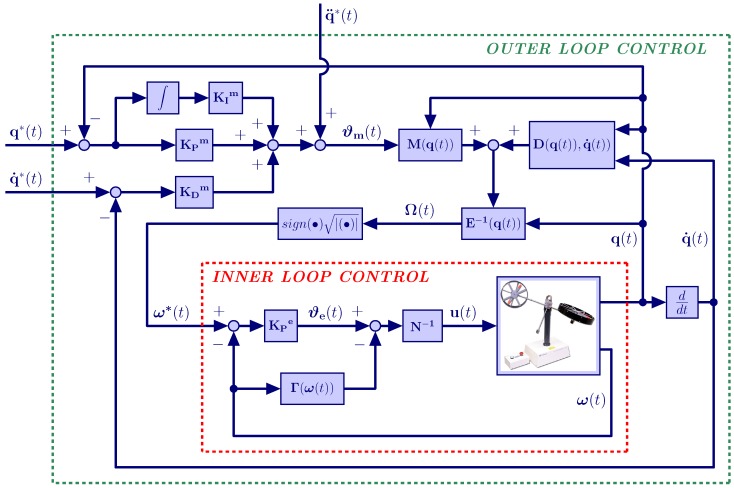
Robust decentralized nonlinear control scheme for the TRMS.

Among the advantages of this control scheme are: (a) the robust nonlinear controller design procedure is simplified to a great extent, since it allows one to design the multivariate inner loop in an independent manner from the multivariate outer loop, thus dividing the control design process into two much simpler design processes; (b) this scheme can be more easily and safely implemented than the standard controllers used in the control of the TRMS platform, which involve closing a single loop, because the nested control loops proposed in this work are sequentially implemented, first closing the inner loop, which exhibits a very high relative stability in the presence of system uncertainties, external disturbances and noisy corruptions, and later closing the outer loop, which is more prone to becoming unstable, but for which the risk of exhibiting unstable motions has been significantly reduced by previously having closed the inner loop; (c) the disturbances affecting the secondary or inner loop are effectively compensated before they affect the main process output, thereby improving the stability of the system; (d) the closing of the control loop around the secondary part of the process reduces the phase lag seen by the primary or outer controller, resulting in increased speed of response; (e) the cascade control scheme is not strongly sensitive to modeling errors, although large errors could lead to oscillations or instability in one of the feedback controllers; (f) any variation in the static gain of the secondary part of the process is compensated by its own tie; (g) the use of this scheme can dramatically improve the performance of control strategies, reducing both the maximum deviation and the integral error for disturbance responses. In the scheme shown in Figure 5, the outer loop controller generates an auxiliary command reference vector $\omega^{*}\left(t\right)={\left[\omega_{m}^{*}\left(t\right),\omega_{t}^{*}\left(t\right)\right]}^{T}$ for the velocities of the propellers on the basis of the tracking objective for the vector of generalized coordinates: $q\left(t\right)={\left[\psi \left(t\right),\phi \left(t\right)\right]}^{T}$. The inner loop controller takes the command vector signal generated by the outer loop $\omega^{*}\left(t\right)$ as its reference for the inner loop propeller velocity control system. The different parts of the proposed control scheme are explained next.

### 3.1. Inner Loop Control

The inner loop control is designed to calculate the required values for the input control voltages of the motors in the MATLAB/Simulink^®^ environment, $u\left(t\right)={\left[u_{m}\left(t\right),u_{t}\left(t\right)\right]}^{T}$, in order to reduce and eliminate the difference between the vector of angular velocities of the propellers of the TRMS, $\omega \left(t\right)={\left[\omega_{m}\left(t\right),\omega_{t}\left(t\right)\right]}^{T}$, and the reference vector of these angular velocities, $\omega^{*}\left(t\right)={\left[\omega_{m}^{*}\left(t\right),\omega_{t}^{*}\left(t\right)\right]}^{T}$, which is the output of the outer loop. In this sense, the feedback multivariate control input, $u\left(t\right)={\left[u_{m}\left(t\right),u_{t}\left(t\right)\right]}^{T}$, is synthesized as a nonlinear input transformation and classical proportional controller with a nonlinear cancellation vector:

(42) $$u\left(t\right)=N^{-1}\left[\vartheta_{e}\left(t\right)-\Gamma \left(\omega \left(t\right)\right)\right]$$

in which N and Γ(ω(t)) are defined in Equations (12) and (13), respectively, and $\vartheta_{e}\left(t\right)={\left[\vartheta_{m}\left(t\right),\vartheta_{t}\left(t\right)\right]}^{T}$ represents a vector of auxiliary control inputs, given by:

(43) $$\vartheta_{e}\left(t\right)=\dot{\omega }\left(t\right)=-K_{P}^{e}\left[\omega \left(t\right)-\omega^{*}\left(t\right)\right]$$

where $K_{P}^{e}\in R^{2\times 2}$ is a constant diagonal positive definitive matrix that represents the design elements of a vector-valued classical proportional controller.

The closed loop tracking error vector, $e_{\omega }\left(t\right)=\omega \left(t\right)-\omega^{*}\left(t\right)$, for the electrical part is obtained after substituting Expression (42) in the dynamic model of the electrical part of the system in Equation (11), yielding the following expression:

(44) $$\dot{\omega }\left(t\right)+K_{P}^{e}e_{\omega }\left(t\right)=0$$

The controller design matrix $K_{P}^{e}$ is designed so as to render the following 2×2 complex-valued diagonal matrix, $p_{c}^{e}\left(s\right)$, defined as:

(45) $$p_{c}^{e}\left(s\right)=I^{2\times 2}s+K_{P}^{e}$$

as first degree Hurwitz polynomials with the desired roots located in the left half of the complex plane in order to achieve the convergence of the tracking error dynamics to a small vicinity around the origin of the error phase space. In particular, the constant controller gain matrix $K_{P}^{e}$ of the closed loop characteristic polynomial is determined by means of a term by term comparison with the following desired Hurtwitz 2×2 diagonal matrix:

(46) $$p_{c_{d}}^{e}\left(s\right)=I^{2\times 2}s+p_{c}^{e}$$

where $p_{c}^{e}\in R^{2\times 2}$ is a diagonal positive definite matrix, which represents the desired position of the poles in closed loop. Therefore, the design controller gain is given by:

(47) $$K_{P}^{e}=p_{c}^{e}$$

Finally, to conclude the description of the inner loop control, we highlight again that the design parameters are selected for the sake of making the dynamics of the inner loop control much faster than the outer loop dynamics, this way ensuring the functioning of the cascade controller [24]. The secondary controller must be relatively quick so that it attenuates a disturbance before the disturbance affects the primary controlled variable. A general guideline is that the secondary one should be three-times faster than the primary [25]. It should be noted that the cascade strategy has to be tuned in a sequential manner. In this procedure, the inner loop control should be tuned first, because the secondary controller or inner loop affects the open-loop dynamics of the primary or outer loop. Thereby, and in order to tune the parameters in the inner loop control, which are the gains of the proportional controller defined in matrix $K_{P}^{e}$, the primary controller will be disconnected, i.e., the cascade should be open, and then, the electrical controller will be tuned in a conventional manner, which involves a plant experiment, initial tuning calculation and fine-tuning based on a closed-loop dynamic response.

### 3.2. Outer Loop Control

The objective of the outer loop control is to determine the required values for the angular velocities of the main and tail rotors, i.e., the reference vector for the angular velocities, which is the reference input of the inner loop, $\omega^{*}\left(t\right)={\left[\omega_{m}^{*}\left(t\right),\omega_{t}^{*}\left(t\right)\right]}^{T}$, in order to eliminate the difference between the generalized coordinates of the TRMS, $q\left(t\right)={\left[\psi (t),\phi (t)\right]}^{T}$, and the reference trajectories for these coordinates, $q^{*}\left(t\right)={\left[\psi^{*}\left(t\right),\phi^{*}\left(t\right)\right]}^{T}$. To achieve this goal, the following multivariate nonlinear feedback control input vector, Ω(t), is synthesized as a nonlinear input transformation and a proportional-integral-derivative (PID) controller with a nonlinear cancellation vector:

(48) $$\Omega \left(t\right)=E^{-1}\left(q\left(t\right)\right)\left[M\left(q\left(t\right)\right)\vartheta_{m}\left(t\right)+D\left(q\left(t\right),\dot{q}\left(t\right)\right)\right]$$

where $\vartheta_{m}\left(t\right)={\left[\vartheta_{\psi }\left(t\right),\vartheta_{\phi }\left(t\right)\right]}^{T}$ represents a vector of auxiliary control variables, given by:

(49) $$\vartheta_{m}\left(t\right)=\ddot{q}\left(t\right)={\ddot{q}}^{*}\left(t\right)-K_{D}^{m}\left(\dot{q}\left(t\right)-{\dot{q}}^{*}\left(t\right)\right)-K_{P}^{m}\left(q\left(t\right)-q^{*}\left(t\right)\right)-K_{I}^{m}\int \left(q\left(t\right)-q^{*}\left(t\right)\right)$$

where $K_{D}^{m}$, $K_{P}^{m}$ and $K_{I}^{m}\in R^{2\times 2}$ are diagonal positive definitive matrices that represent the design elements of a vector-valued classical proportional-integral-derivative multivariate controller.

The closed loop tracking error vector, $e_{q}\left(t\right)=q\left(t\right)-q^{*}\left(t\right)$, for the mechanical part is obtained after substituting Expression (48) in the simplified model of the mechanical part in Equation (39), yielding the following expression:

(50) $$e_{q}^{\left(3\right)}\left(t\right)+K_{D}^{m}{\ddot{e}}_{q}\left(t\right)+K_{P}^{m}{\dot{e}}_{q}\left(t\right)+K_{I}^{m}e_{q}\left(t\right)=0$$

In order to achieve the convergence of the tracking error dynamics to a small vicinity around the origin of the tracking error phase space, the controller design matrices $K_{D}^{m}$, $K_{P}^{m}$ and $K_{I}^{m}$ are chosen in such a manner that all non-zero components of the 2×2 complex valued diagonal matrix, $p_{c}^{m}\left(s\right)$, defined as,

(51) $$p_{c}^{m}\left(s\right)=I^{2\times 2}s^{3}+K_{D}^{m}s^{2}+K_{P}^{m}s+K_{I}^{m}$$

are all third degree Hurwitz polynomials whose roots are located sufficiently far into the left half on the complex plane. The stability of Expression (51) can be studied by using the Routh–Hurwitz criterion. Bearing in mind that the set of design matrices $K_{P}^{m}$, $K_{D}^{m}$ and $K_{I}^{m}$ are diagonal, the stability of each error variable $e_{q}\left(t\right)={\left[e_{\psi }\left(t\right);e_{\phi }\left(t\right)\right]}^{T}={\left[\psi \left(t\right)-\psi^{*}\left(t\right);\phi \left(t\right)-\phi^{*}\left(t\right)\right]}^{T}$ can be studied in an independent manner. After applying the Routh–Hurwitz criterion, one obtains the following stability conditions: (i) $K_{D_{i}}^{m},K_{P_{i}}^{m}>0$; and (ii) $0<K_{I_{i}}^{m}<K_{D_{i}}^{m}\cdot K_{P_{i}}^{m}$ for i=ψ,ϕ. After considering the previous stability restrictions, the constant controller gains $K_{D}^{m}$, $K_{P}^{m}$ and $K_{I}^{m}$ of the closed loop characteristic polynomial are determined by using a term by term comparison with the following desired Hurtwitz 2×2 complex-valued diagonal matrix:

(52) $$p_{c_{d}}^{m}\left(s\right)=\left(I^{2\times 2}s+p_{c}^{m}\right)\left(I^{2\times 2}s^{2}+2\zeta_{c}^{m}\omega_{c}^{m}s+{\left(\omega_{c}^{m}\right)}^{2}\right)$$

where $p_{c}^{m}$, $\zeta_{c}^{m}$ and $\omega_{c}^{m}\in R^{2\times 2}$ are diagonal positive definite matrices. Therefore, the design controller gains are given by:

(53) $$\begin{array}{ccc} K_{D}^{m} & = & 2\zeta_{c}^{m}\omega_{c}^{m}+p_{c}^{m} \end{array}$$

(54) $$\begin{array}{ccc} K_{P}^{m} & = & {\left(\omega_{c}^{m}\right)}^{2}+2\zeta_{c}^{m}\omega_{c}^{m}p_{c}^{m} \end{array}$$

(55) $$\begin{array}{ccc} K_{I}^{m} & = & p_{c}^{m}{\left(\omega_{c}^{m}\right)}^{2} \end{array}$$

Finally, the necessary angular velocity vector values, $\omega^{*}\left(t\right)={\left[\omega_{m}^{*}\left(t\right),\omega_{t}^{*}\left(t\right)\right]}^{T}$, are obtained from the input control vector, $\Omega \left(t\right)={\left[\omega_{m}\left|\omega_{m}\right|,\omega_{t}\left|\omega_{t}\right|\right]}^{T}$, by performing the following operation:

(56) $$\omega^{*}\left(t\right)=\left[\begin{array}{c} \omega_{m}^{*}\left(t\right)\\ \omega_{t}^{*}\left(t\right) \end{array}\right]=\left[\begin{array}{c} sign\left(\omega_{m}\left|\omega_{m}\right|\right)\sqrt{\left|\omega_{m}\left|\omega_{m}\right|\right|}\\ sign\left(\omega_{t}\left|\omega_{t}\right|\right)\sqrt{\left|\omega_{t}\left|\omega_{t}\right|\right|} \end{array}\right]$$

## 4. Experimental Section

This section describes the experiments carried out to verify the effectiveness of the proposed control algorithm. In the following subsections, we briefly explain the experimental platform and the software tools, and after that, we illustrate the experimental results on the real platform, including a comparison with other control algorithms in terms of both stabilization and trajectory tracking task performance.

### 4.1. Experimental Setup

The implementation of the designed robust decentralized controller is carried out by using the following equipment:

- A twin rotor MIMO system provided by Feedback Instruments^®^ (see Figure 1 and [9]).
- A PC operating in a Windows^®^ environment using software tools from *MathWorks^®^ Inc* (MATLAB^®^, Simulink, Control Toolbox, Real Time Workshop^®^ (RTW), Real Time Windows Target^®^ (RTWT)) and Visual $C^{++}$ Professional^®^.
- The real TRMS is connected to the computer by means of an Advantech^®^ PCI1711 card, which is accessible in the MATLAB/Simulink^®^ environment through the Real-Time Toolbox^®^.
- The control signals in the MATLAB/Simulink^®^ environment consist of two input voltages (in the range [−2.5,2.5] *V*) for the two DC motors A-max 26 provided by Maxon Motor^®^.
- The vector of generalized coordinates, $q\left(t\right)={\left[\psi \left(t\right),\phi \left(t\right)\right]}^{T}$, are measured by using two HCTL 2016 digital encoders provided by Agilent Technologies^®^, and the angular velocity vector $\omega \left(t\right)={\left[\omega_{m}\left(t\right),\omega_{t}\left(t\right)\right]}^{T}$ is measured by using two DC-Tacho DCT 22 provided by Maxon Motor^®^.
- The sampling rate for the controlled system is 0.002 s.

**Figure 6:**
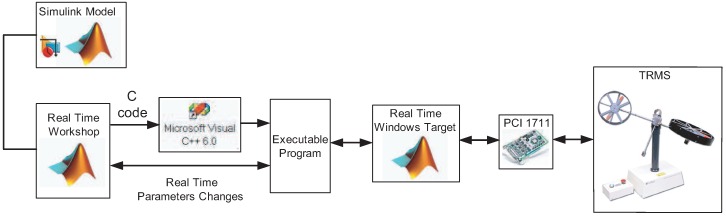
Control system development flow diagram.

On the other hand, the executable file for the proposed control scheme is achieved by performing the following steps (see Figure 6): MATLAB^®^ acts as the application host environment, in which the other MathWorks^®^ products run, and Simulink^®^ provides a well-structured graphical interface for the implementation of the proposed nonlinear control scheme. Real Time Workshop^®^ automatically builds a $C^{++}$ source program from the Simulink Model. The $C^{++}$ Compiler^®^ compiles and links the code created by Real Time Workshop^®^ to produce an executable program. Real Time Windows Target^®^ communicates with the executable program acting as the control program and interfaces with the TRMS through the PCI1711 card. Real Time Windows Target^®^ controls the two-way data, or signal flow, to and from the model (which is now an executable program), and to and from the PCI1711 card. The advantage of this approach is that the designer only needs to model the process, using the graphical tools available in Simulink^®^, without having to worry about the mechanics of communication to and from the TRMS.

### 4.2. Experimental Results

This subsection discusses the experiments carried out to verify the efficiency of the control strategy proposed in Section 3 in the following aspects: (a) robustness with regard to large initial errors; (b) quick convergence of the tracking errors to a small neighborhood of zero; (c) smooth transient responses; (d) low control effort; (e) robustness with regard to modeling errors. In the trials, the desired reference trajectories for the pitch (*ψ*) and the yaw (*ϕ*) angles have been selected in order to obtain a complex and challenging trajectory for the TRMS, which allows one to show the main characteristics and excellent performance of the proposed control scheme, but avoiding at the same time the saturation of the actuators of the laboratory platform. This reference trajectory is defined by the following expression:

(57) $$q^{*}\left(t\right)=\left[\begin{array}{c} \psi^{*}\left(t\right)\\ \phi^{*}\left(t\right) \end{array}\right]=\left[\begin{array}{c} A_{0_{\psi }}+A_{1_{\psi }}\left(2sin\left(\omega_{1_{\psi }}t\right)+sin\left(\omega_{2_{\psi }}t\right)\right)\\ A_{1_{\phi }}sin\left(\omega_{1_{\phi }}t\right)+A_{2_{\phi }}\left(sin\left(\omega_{2_{\phi }}t\right)+sin\left(\omega_{3_{\phi }}t\right)\right) \end{array}\right]$$

where $q^{*}\left(t\right)={\left[\psi^{*}\left(t\right),\phi^{*}\left(t\right)\right]}^{T}$ is the reference trajectory vector of the generalized coordinates, and the values of the above constants are given by:

(58) $$\begin{array}{cccccc} A_{0_{\psi }} & =0.4\;rad; & \; & \; & \omega_{1_{\psi }} & =0.0785\;rad/s;\\ A_{1_{\psi }} & =0.1\;rad; & \; & \; & \omega_{2_{\psi }} & =0.0157\;rad/s;\\ A_{1_{\phi }} & =0.8\;rad; & \; & \; & \omega_{1_{\phi }} & =0.157\;rad/s;\\ A_{2_{\phi }} & =0.3\;rad; & \; & \; & \omega_{2_{\phi }} & =0.0785\;rad/s;\\ & & \; & \; & \omega_{3_{\phi }} & =0.0157\;rad/s; \end{array}$$

In order to demonstrate the exponential convergence of the desired trajectories, and the robustness with respect to large initial errors, the initial position of the TRMS is defined as $q_{0}\left(t\right)={\left[0,0\right]}^{T}$, which represents a different value than the initial position of the reference trajectory vector $q^{*}\left(t\right)$. With regard to the parameters of the plant used in the experimentation, the values of which are presented in Table 1, Table 2 and Table 3, we have to highlight that the discrepancies in the model due to modeling errors are around 5%, as a consequence of the difficulty involved in adequately modeling all of the dynamics terms. The small errors observed in the dynamics identification trials, which have been performed in our research, are compensated by the action of the proposed control scheme. With the use of an integral action on the outer loop, eliminating the possible steady state errors is achieved.

On the other hand, the design of the proposed nonlinear control scheme and the choice of the values of the gain vectors, which are tuned according to the procedure explained in Section 3, have been done in order to achieve a control as fast as possible, but avoiding possible saturations of the input voltages of the motors in the MATLAB/Simulink^®^ environment, which occur at ±2.5 V. The summary of the procedure carried out to tune the designer parameters is explained next. Firstly, the inner loop control has been tuned using the model of the electrical part of the TRMS by means of numerical simulations. In this first stage, the parameters of the proportional controller have been tuned in order to achieve the fast dynamics of the inner loop. In other words, the aim is to achieve a quick convergence of the closed loop tracking error vector, $e_{\omega }\left(t\right)$, to a small vicinity around the origin of the tracking error phase space. Secondly, we have assumed the dynamics of the inner loop to be equal to $I^{2\times 2}$, and then, we have tuned, again by means of numerical simulations, the parameters of the PID controller in the outer loop. Finally, the values obtained in the simulations have been slightly adjusted in the experimental trials with the laboratory platform. Thereby, for the inner loop controller, the values of the desired Hurtwitz 2×2 complex diagonal matrix for the controller are $p_{c}^{e}\left(s\right)=diag\left(12.0,9.0\right)$, and for the outer loop controller, the values of the matrices of the desired Hurtwitz polynomial vector for the feedback controller are $p_{c}^{m}=diag\left(1.0,1.0\right)$, $\zeta_{c}^{m}=diag\left(1.5,1.5\right)$ and $\omega_{c}^{m}=diag\left(2.0,1.8\right)$. More details about how to tune controllers based on a cascade scheme can be consulted in some reference works [25,26,27,28].

**Figure 7:**
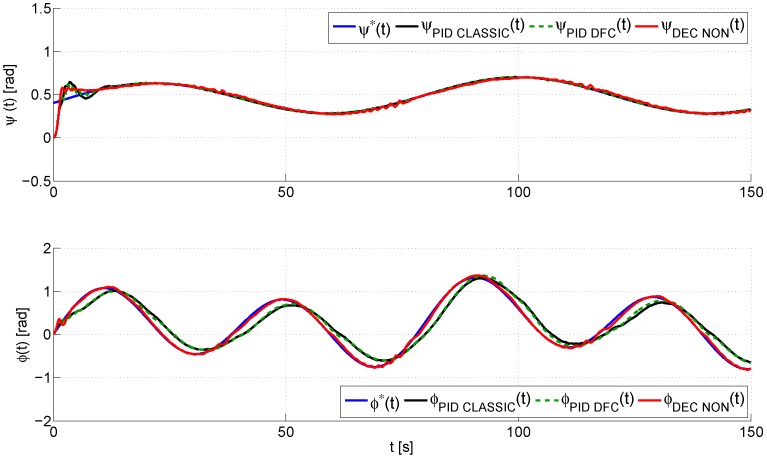
Real and desired evolution trajectories of the vector of the generalized coordinates of the TRMS, $q\left(t\right)={\left[\psi \left(t\right),\phi \left(t\right)\right]}^{T}$.

**Figure 8:**
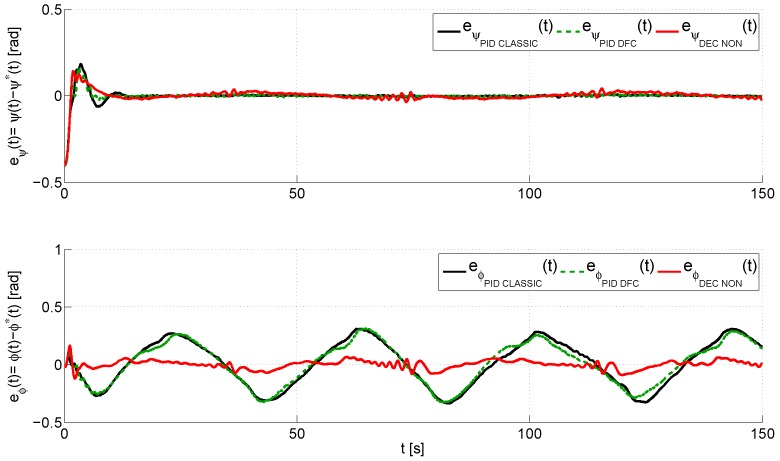
Evolution of the error vector of generalized coordinates of the TRMS, $e_{q}\left(t\right)=q\left(t\right)-q^{*}\left(t\right)={\left[\psi \left(t\right)-\psi^{*}\left(t\right),\phi \left(t\right)-\phi^{*}\left(t\right)\right]}^{T}$.

**Figure 9:**
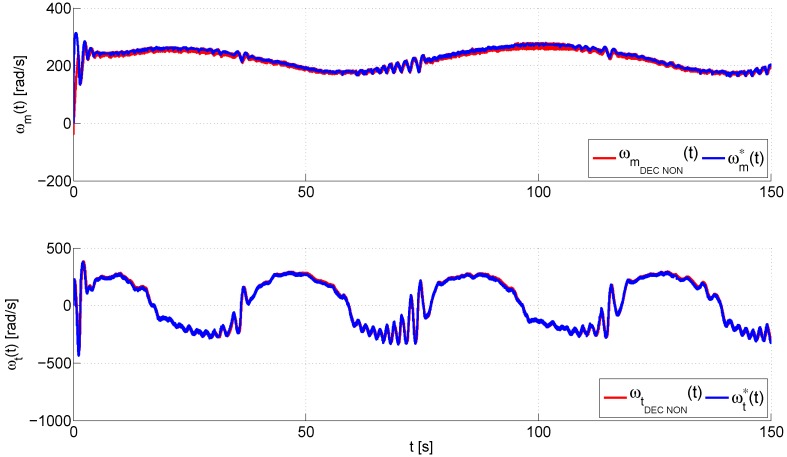
Real and desired evolution trajectories of the angular velocity vector, $\omega^{*}\left(t\right)={\left[\omega_{m}^{*}\left(t\right),\omega_{t}^{*}\left(t\right)\right]}^{T}$ and $\omega \left(t\right)={\left[\omega_{m}\left(t\right),\omega_{t}\left(t\right)\right]}^{T}$.

**Figure 10:**
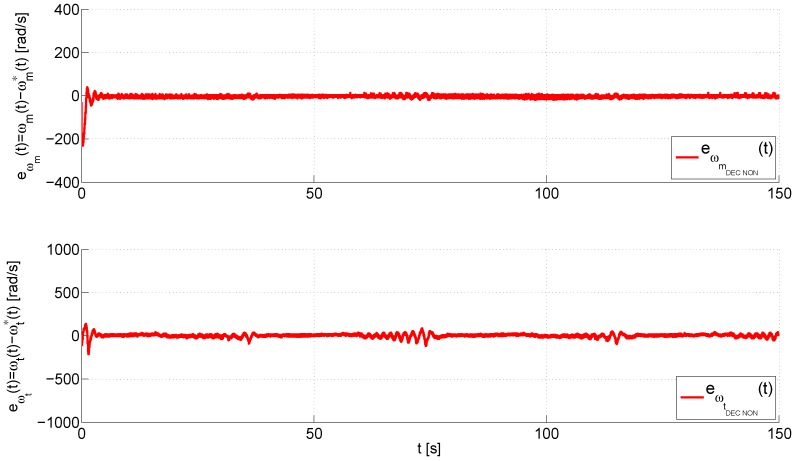
Evolution of the angular velocity error vector, $e_{\omega }\left(t\right)=\omega \left(t\right)-\omega^{*}\left(t\right)=[\omega_{m}\left(t\right)-\omega_{m}^{*}\left(t\right)$, $\omega_{t}\left(t\right)-\omega_{t}^{*}{\left(t\right)]}^{T}$.

**Figure 11:**
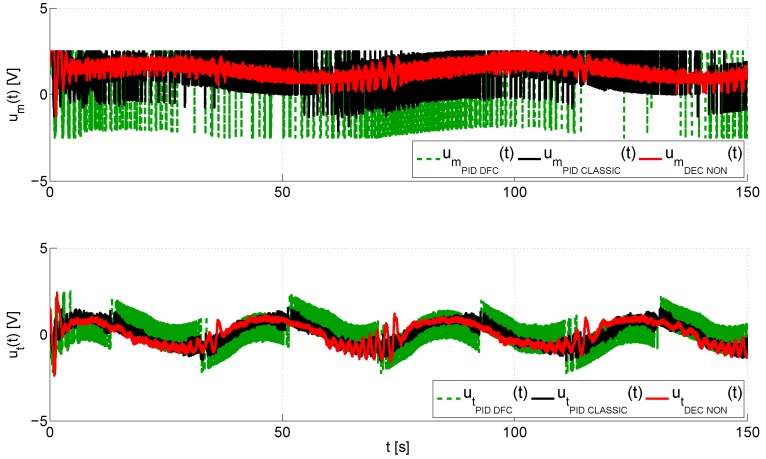
Evolution of the input voltage vector, $u\left(t\right)={\left[u_{m}\left(t\right),u_{t}\left(t\right)\right]}^{T}$, in the MATLAB/Simulink^®^ environment.

In the following lines, we discuss the performance of the proposed decentralized control scheme, which is shown in the next graphs (Figure 7, Figure 8, Figure 9, Figure 10 and Figure 11), where, in order to show the improvements of this design, we shall also compare the experimental results obtained using the proposed control (denoted in the graphs as decentralized nonlinear control (DEC NON)), a standard PID control [29] (denoted in the graphs as PID CLASSIC) and a PID control with a derivative filter coefficient [19] (denoted in the graphs as PID DFC). Figure 7 illustrates a comparison between the desired trajectory, $q^{*}\left(t\right)={\left[\psi^{*}\left(t\right),\phi^{*}\left(t\right)\right]}^{T}$, and the real trajectory of the TRMS, $q\left(t\right)={\left[\psi (t),\phi (t)\right]}^{T}$. This graph shows that the three algorithms are robust with regard to large initial errors. However, the proposed decentralized control scheme has the smoothest transient response and the best performance in trajectory tracking, as can also be observed in Figure 8, which shows, for each control, the closed loop tracking error vector, $e_{q}\left(t\right)=q\left(t\right)-q^{*}\left(t\right)={\left[\psi \left(t\right)-\psi^{*}\left(t\right),\phi \left(t\right)-\phi^{*}\left(t\right)\right]}^{T}$. The proposed decentralized controller has a closed loop tracking error vector that remains bounded within a vicinity of radius ${\left[0.04,0.10\right]}^{T}\;rad$, while the standard PID and the PID with derivative filter have error vectors bounded in ${\left[0.02,0.35\right]}^{T}\;rad$ and ${\left[0.02,0.33\right]}^{T}\;rad$, respectively. Therefore, although the three control algorithms achieve a quick convergence of the tracking error to a small neighborhood of zero, the proposed control scheme presents the smallest error.

On the other hand, the performance of the inner control loop is shown in Figure 9, which illustrates a comparison between the angular velocity vector, $\omega^{*}\left(t\right)={\left[\omega_{m}^{*}\left(t\right),\omega_{t}^{*}\left(t\right)\right]}^{T}$, obtained from the output of the outer loop, and the real magnitudes of the angular velocity vector, $\omega \left(t\right)={\left[\omega_{m}\left(t\right),\omega_{t}\left(t\right)\right]}^{T}$. Again, the proposed control has a smooth transient response and a fast convergence of the tracking error to a neighborhood near to zero as evidenced in Figure 10, which shows, for each control, that the angular velocity error vector, $e_{\omega }\left(t\right)=\omega \left(t\right)-\omega^{*}\left(t\right)={\left[\omega_{m}\left(t\right)-\omega_{m}^{*}\left(t\right),\omega_{t}\left(t\right)-\omega_{t}^{*}\left(t\right)\right]}^{T}$, remains bounded in ${\left[20,120\right]}^{T}\;rad/s$. Finally, the input voltage vectors in the MATLAB/Simulink^®^ environment, $u\left(t\right)={\left[u_{m}\left(t\right),u_{t}\left(t\right)\right]}^{T}$, are shown in Figure 11. This graph illustrates that the smallest control input effort is provided by the proposed control scheme, which furthermore presents a smooth evolution of the input voltage vector without saturations unlike both PID controls, the standard PID and the PID with derivative filter coefficient. As you may observe at the top of this figure, both PID controls cause the saturation of the control signal of the main rotor, which occurs at ±2.5 V, for long periods of time during the trials. These saturations cause a worse performance of each one of these controllers in comparison with the proposed control scheme.

Additionally, the performances of the control methods have been measured in terms of the integral squared tracking error, $ISE=\int_{t_{A}}^{t_{B}}e_{q}{\left(t\right)}^{T}e_{q}\left(t\right)$d$t=\int_{t_{A}}^{t_{B}}\left(e_{\psi }{\left(t\right)}^{2}+e_{\phi }{\left(t\right)}^{2}\right)$d*t*, the integral absolute tracking error, $IAE=\int_{t_{A}}^{t_{B}}\left(\right|e_{\psi }\left(t\right)|+|e_{\phi }\left(t\right)\left|\right)$d*t*, and the integral time absolute tracking error, $ITAE=\int_{t_{A}}^{t_{B}}t(|e_{\psi }\left(t\right)|+|e_{\phi }\left(t\right)\left|\right)$d*t*, where $t_{A}=0\;s$ and $t_{B}=150\;s$ denote the initial and final time of the simulation, and $e_{q}\left(t\right)={\left[e_{\psi }\left(t\right),e_{\phi }\left(t\right)\right]}^{T}={\left[\psi \left(t\right)-\psi^{*}\left(t\right),\phi \left(t\right)-\phi^{*}\left(t\right)\right]}^{T}$ is the closed loop tracking error vector. The *ISE* and the *IAE* criteria will treat all of the tracking errors in a uniform manner. However, the *ITAE* criterion, as time appears as a factor, will heavily penalize errors that occur late in time, but ignore errors that occur early in time. The results achieved are illustrated in Table 4, showing the best performance of the proposed decentralized control scheme (DEC NON) in comparison to the other conventional controls (PID CLASSIC and PID DFC). Both PID controls show a similar behavior and have a worse performance when they are compared to the proposed control method.

To sum up, the experimental results show a better performance of the proposed decentralized control scheme against the other control laws. The proposed control law illustrates a better performance in the following aspects: (1) robustness in relation to large initial errors with a smooth transient response; (2) better tracking of the reference trajectories; (3) quick convergence of the tracking errors to the smallest neighborhood of zero; (4) less control effort; and (5) the absence of saturations in the input control voltages.

## 5. Conclusions

In this study, we have successfully designed a novel robust nonlinear multivariate decentralized control scheme for the underactuated and nonlinear twin rotor MIMO system (TRMS) laboratory platform. This control system is based on decoupling the electrical from the mechanical dynamics and the use of two nested nonlinear multivariate loops. The inner loop is designed as a nonlinear input transformation and classical proportional controller with a nonlinear cancellation vector and is responsible for the stabilization and tracking of the vector of angular velocities of the propellers of the TRMS. The outer loop control is designed as a nonlinear input transformation, a proportional-integral-derivative (PID) linear action and nonlinear compensation vector, which determines the required values for the reference velocities in order to achieve the elimination of the difference between the generalized coordinates of the TRMS and the reference trajectories for these. This independence in the design of the control loops is possible thanks to having made the dynamics of the inner loop much faster than the dynamics of the mechanical part. This control system is very simple and allows the platform to be perfectly stabilized and positioned in space. Additional advantages of this control approach are: (a) simplification of the control design procedure due to the design of two much simpler dynamics, which are controlled separately; (b) this scheme can be more easily and safely implemented than the standard controllers used in the control of the TRMS platform, which involve closing a single loop, because the nested control loops proposed in this work are sequentially implemented, first by closing the inner loop, which exhibits a very high relative stability in the presence of system uncertainties, external disturbances and noisy corruptions, and later through closing the outer loop, which is more prone to becoming unstable, but whose risk of exhibiting unstable motions has been significantly reduced by having previously closed the inner loop. The experimental tests carried out, in order to verify the performance of the proposed decentralized controller, show not only the accurate tracking of the reference trajectories, but also the better performance of the proposed control compared to the other two conventional controllers. The robustness in regards to large initial errors and possible modeling errors, the quick convergence to a small neighborhood of zero and the smooth transient response with a low control effort are the main features of the proposed design.

## Figures and Tables

**Table 1:** Dynamic model of the TRMS: electrical parameters.

Symbol	Parameter	Value	Units
***Parameters of the Main Rotor***			
$k_{v_{m}}$	Motor velocity constant	0.0202	$V\cdot rad^{-1}\cdot s$
$R_{m}$	Motor armature resistance	8	Ω
$L_{m}$	Motor armature inductance	$0.86\times 10^{-3}$	*H*
$k_{t_{m}}$	Electromagnetic constant torque motor	0.0202	$N\cdot m\cdot A^{-1}$
$k_{u_{m}}$	Coefficient linear relationship interface circuit	8.5	−
$C_{Q_{m}}^{+}$	Load factor ($\omega_{m}\geq 0$)	$2.695\times 10^{-7}$	$N\cdot m\cdot s^{2}\cdot rad^{-2}$
$C_{Q_{m}}^{-}$	Load factor ($\omega_{m}<0$)	$2.46\times 10^{-7}$	$N\cdot m\cdot s^{2}\cdot rad^{-2}$
$f_{v_{m}}$	Viscous friction coefficient	$3.89\times 10^{-6}$	$N\cdot m\cdot rad^{-1}\cdot s$
$I_{m1}$	Moment of inertia about the axis of rotation	$1.05\times 10^{-4}$	$kg\cdot m^{2}$
$c_{e_{m}}$	Electrical time constant ($L_{m}/R_{m}$)	$1.075\times 10^{-4}$	*s*
$c_{m_{m}}$	Mechanical time constant ($I_{m1}R_{m}/k_{t_{m}}k_{v_{m}}$)	2.058	*s*
***Parameters of the Tail Rotor***			
$k_{v_{t}}$	Motor velocity constant	0.0202	$V\cdot rad^{-1}\;s$
$R_{t}$	Motor armature resistance	8	Ω
$L_{t}$	Motor armature inductance	$0.86\times 10^{-3}$	*H*
$k_{t_{t}}$	Electromagnetic constant torque motor	0.0202	$N\cdot m\cdot A^{-1}$
$k_{u_{t}}$	Coefficient linear relationship interface circuit	6.5	−
$C_{Q_{t}}$	Load factor	$1.164\times 10^{-8}$	$N\cdot m\cdot s^{2}\cdot rad^{-2}$
$f_{v_{t}}$	Viscous friction coefficient	$1.715\times 10^{-6}$	$N\cdot m\cdot rad^{-1}\cdot s$
$I_{t1}$	Moment of inertia about the axis of rotation	$2.1\times 10^{-5}$	$kg\cdot m^{2}$
$c_{e_{t}}$	Electrical time constant ($L_{t}/R_{t}$)	$1.075\times 10^{-4}$	*s*
$c_{m_{t}}$	Mechanical time constant ($I_{t1}R_{t}/k_{t_{t}}k_{v_{t}}$)	0.4117	*s*

**Table 2:** Dynamic model of the TRMS: mechanical parameters.

Symbol	Parameter	Value	Units
$l_{t}$	Length of the tail part of the free-free beam	0.282	m
$l_{m}$	Length of the main part of the free-free beam	0.246	m
$l_{b}$	Length of the counterbalance beam	0.290	m
$l_{cb}$	Distance between the counterweight and the joint	0.276	m
$r_{ms}$	Radius of the main shield	0.155	m
$r_{ts}$	Radius of the tail shield	0.1	m
*h*	Length of the pivoted beam	0.06	m
$m_{tr}$	Mass of the tail DC motor and tail rotor	0.221	kg
$m_{mr}$	Mass of the main DC motor and main rotor	0.236	kg
$m_{cb}$	Mass of the counterweight	0.068	kg
$m_{t}$	Mass of the tail part of the free-free beam	0.015	kg
$m_{m}$	Mass of the main part of the free-free beam	0.014	kg
$m_{b}$	Mass of the counterbalance beam	0.022	kg
$m_{ts}$	Mass of the tail shield	0.119	kg
$m_{ms}$	Mass of the main shield	0.219	kg
$m_{h}$	Mass of the pivoted beam	0.01	kg

**Table 3:** Dynamic model of the TRMS: parameters of the pitch and yaw movements.

Symbol	Parameter	Value	Units
***Parameters of the Pitch movement***			
$C_{T_{m}}^{+}$	Thrust torque coefficient of the main rotor ($\omega_{m}\geq 0$)	$1.53\times 10^{-5}$	$N\cdot s^{2}\cdot rad^{-2}$
$C_{T_{m}}^{-}$	Thrust torque coefficient of the main rotor ($\omega_{m}<0$)	$8.8\times 10^{-6}$	$N\cdot s^{2}\cdot rad^{-2}$
$C_{R_{t}}$	Load torque coefficient of the tail rotor	$9.7\times 10^{-8}$	$N\cdot m\cdot s^{2}\cdot rad^{-2}$
$f_{v_{\psi }}$	Viscous friction coefficient	0.0024	$N\cdot m\cdot s\cdot rad^{-1}$
$f_{c_{\psi }}$	Coulomb friction coefficient	$5.69\times 10^{-4}$	N·m
$k_{t}$	Coefficient of the inertial counter torque due to change in $\omega_{t}$	$2.6\times 10^{-5}$	$N\cdot m\cdot s^{2}\cdot rad^{-1}$
***Parameters of the Yaw movement***			
$C_{T_{t}}^{+}$	Thrust torque coefficient of the tail rotor ($\omega_{t}\geq 0$)	$3.25\times 10^{-6}$	$N\cdot s^{2}\cdot rad^{-2}$
$C_{T_{t}}^{-}$	Thrust torque coefficient of the tail rotor ($\omega_{t}<0$)	$1.72\times 10^{-6}$	$N\cdot s^{2}\cdot rad^{-2}$
$C_{R_{m}}^{+}$	Load torque coefficient of the main rotor ($\omega_{m}\geq 0$)	$4.9\times 10^{-7}$	$N\cdot m\cdot s^{2}\cdot rad^{-2}$
$C_{R_{m}}^{-}$	Load torque coefficient of the main rotor ($\omega_{m}<0$)	$4.1\times 10^{-7}$	$N\cdot m\cdot s^{2}\cdot rad^{-2}$
$f_{v_{\phi }}$	Viscous friction coefficient	0.03	$N\cdot m\cdot s\cdot rad^{-1}$
$f_{c_{\phi }}$	Coulomb friction coefficient	$3\times 10^{-4}$	N·m
$c_{c}$	Coefficient of the elastic force torque created by the cable	0.016	$N\cdot m\cdot rad^{-1}$
$\phi_{0}$	Constant for the calculation of the torque of the cable	0	rad
$k_{m}$	Coefficient of the inertial counter torque due to change in $\omega_{m}$	$2\times 10^{-4}$	$N\cdot m\cdot s^{2}\cdot rad^{-1}$

**Table 4:** Performance of the control methods.

Control Method	*ISE*	*IAE*	*ITAE*
Robust Decentralized Nonlinear Control (DEC NON)	0.3956	6.6579	435.7
Standard PID control (PID CLASSIC)	5.8275	26.7591	2002.4
PID control with the derivative filter coefficient (PID DFC)	5.0814	24.8175	1834.4

## References

[1] Nonami K., Kendoul F., Suzuki S.T. (2010). Autonomous Flying Robots—Unmanned Aerial Vehicles and Micro Aerial Vehicles.

[2] Espizona T., Dzul A., Llama M. (2013). Linear and Nonlinear Controllers Applied to Fixed-Wing UAV. Int. J. Adv. Robot. Syst..

[3] Fernández-Caballero A., Belmonte L.M., Morales R., Somolinos J.A. (2015). Generalized Proportional Integral Control for an Unmanned Quadrotor System. Int. J. Adv. Robot. Syst..

[4] Alvarenga J., Vitzilaios N.I., Valavanis K.P., Rutherford M.J. (2015). Survey of Unmanned Helicopter Model-Based Navigation and Control Techniques. J. Intell. Robot. Syst..

[5] Ali Z.A., Wang D., Aamir M. (2016). Fuzzy-Based Hybrid Control Algorithm for the Stabilization of a Tri-Rotor UAV. Sensors.

[6] Cabecinhas D., Naldi R., Silvestre C., Cunha R., Marconi L. (2016). Robust Landing and Sliding Maneuver Hybrid Controller for a Quadrotor Vehicle. IEEE Trans. Control Syst. Technol..

[7] Chen F., Wu Q., Jiang B., Tao G. (2015). A Reconfiguration Scheme for Quadrotor Helicopter via Simple Adaptive Control and Quantum Logic. IEEE Trans. Ind. Electron..

[8] Zheng B., Zhong Y. (2011). Robust Attitude Regulation of a 3-DOF Helicopter Benchmark: Theory and Experiments. IEEE Trans. Ind. Electron..

[9] Feedback Co. (1998). Twin Rotor MIMO System 33-220 User Manual.

[10] Rahideh A., Shaheed M.H., Huigberts H.J.C. (2008). Dynamic Modelling of a TRMS Using Analytical and Empirical Approaches. Control Eng. Pract..

[11] Toha S.F., Latiff I.A., Mohamad M., Tokhi M.O. Parametric modelling of a TRMS using dynamic spread factor particle swarm optimisation. Proceedings of the UKSim 2009: 11th International Conference on Computer Modelling and Simulation.

[12] Tanaka H., Ohta Y., Okimura Y. (2011). A local approach to LPV-identification of a Twin Rotor MIMO System. IFAC Proc. Vol..

[13] Tastemirov A., Lecchini-Visintini A., Morales R.M. Complete Dynamic Model of the TWIN Rotor MIMO System (TRMS) with Experimental Validation. Proceedings of the 39th European Rotorcraft Forum 2013 (ERF 2013).

[14] Chalupa P., Prikryl J., Novák J. (2015). Modelling of Twin Rotor MIMO System. Procedia Eng..

[15] Juang J.-G., Lin R.-W., Liu W.-K. (2008). Comparison of classical control and intelligent control for a MIMO system. Appl. Math. Comput..

[16] Wen P., Lu T.W. (2008). Decoupling control of a twin rotor mimo system using robust deadbeat control technique. IET Control Theory Appl..

[17] Rahideh A., Shaheed M.H. (2012). Constrained output feedback model predictive control for nonlinear systems. Control Eng. Pract..

[18] Jahed M., Farrokhi M. (2013). Robust adaptive fuzzy control of twin rotor MIMO system. Soft Comput..

[19] Kumar-Pandey S., Laxmi V. Control of Twin Rotor MIMO System using PID controller with derivative filter coefficient. Proceedings of the 2014 IEEE Students’ Conference on Electrical, Electronics and Computer Science.

[20] Belmonte L.M., Morales R., Fernández-Caballero A., Somolinos J.A. (2016). A Tandem Active Disturbance Rejection Control for a Laboratory Helicopter with Variable Speed Rotors. IEEE Trans. Ind. Electron..

[21] Alagoz B.B., Ates A., Yeroglu C. (2013). Auto-tuning of PID controller according to fractional-order reference model approximation for DC rotor control. Mechatronics.

[22] Mullhaupt P., Srinivasan B., Levine J., Bonvin D. (2008). Control of the Toycopter Using a Flat Approximation. IEEE Trans. Control Syst. Technol..

[23] Morales R., Feliu V., Jaramillo V. (2012). Position control of very lightweight single-link flexible arms with large payload variations by using disturbance observers. Robot. Auton. Syst..

[24] Son Y.I., Kim I.H., Choi D.S., Shim D. (2015). Robust Cascade Control of Electric Motor Drives Using Dual Reduced-Order PI Observers. IEEE Trans. Ind. Electron..

[25] Marlin T.E. (2000). Process Control, Designing Processes and Control Systems for Dynamic Performance.

[26] Arrieta O., Vilanova R., Balaguer P. (2008). Procedure for cascade control systems design: Choice of suitable PID tunings. Int. J. Comput. Commun. Control.

[27] Alfaro V.M., Vilanova R., Arrieta O. (2009). Robust tuning of Two-Degree-of-Freedom (2-DoF) PI/PID based cascade control systems. J. Process Control.

[28] Veronesi M., Visioli A. (2011). Simultaneous closed-loop automatic tuning method for cascade controllers. IET Control Theory Appl..

[29] Feedback Co. (2008). Twin rotor MIMO system. Control Experiments.

